# Growth performance and bone zinc concentration in broiler chickens with dietary supplementation of organic zinc

**DOI:** 10.5713/ab.25.0665

**Published:** 2025-10-22

**Authors:** Hansol Kim, Tuoying Ao, Jose Soto, Lauren Nolan, Michael Joseph Ford, Sunday Adetayo Adedokun

**Affiliations:** 1 Department of Animal and Food Sciences, University of Kentucky, Lexington, KY, USA; 2 Alltech Inc., Nicholasville, KY, USA

**Keywords:** Broiler, Organic Zinc, Performance, Tibia Zinc

## Abstract

**Objective:**

The objective was to determine the optimal dietary organic zinc (Zn) concentration to maximize growth performance and skeletal Zn deposition in broilers.

**Methods:**

A total of 240 one-d-old broiler chicks were assigned to 5 dietary treatments for a 20-d feeding trial, with 8 cages per treatment. All diets met or exceeded recommended nutrient requirement estimates for broilers, except for Zn. The 5 treatments were as follows: (1) a corn-soybean meal-based diet with 40 mg/kg inorganic Zn from ZnSO_4_·7H_2_O; (2) a corn-soybean meal-based diet without supplemental Zn; (3–5) treatment 2 supplemented with 5, 10, or 20 mg/kg of organic Zn from Zn proteinate. Body weight gain, feed intake, and feed conversion ratio were measured from d 0 to 20. On d 20, tibia samples were collected, and Zn concentration in tibia ash was determined. Orthogonal polynomial contrasts were used to evaluate linear and quadratic responses to increasing supplemental organic Zn. Pairwise contrasts were performed between diets containing 40 mg/kg of inorganic Zn and those containing either 10 or 20 mg/kg of organic Zn. The optimal supplemental level of organic Zn for tibia Zn concentration was estimated using a one-slope broken-line model.

**Results:**

Average daily gain, feed intake, and tibia Zn concentration increased linearly (p<0.05) with increasing organic Zn. Broilers fed 10 mg/kg organic Zn exhibited similar growth to those receiving 40 mg/kg inorganic Zn, but lower tibia Zn concentration (p< 0.001). In contrast, 20 mg/kg organic Zn supported both growth and bone Zn deposition equivalent to the inorganic Zn group. A one-slope broken-line analysis indicated that 16.5 mg/kg organic Zn was the minimum level required to maximize tibia Zn concentration.

**Conclusion:**

Bioplex Zn was more bioavailable than inorganic Zn from Zn sulfate, and 16.5 mg/kg was sufficient to optimize both growth and skeletal Zn deposition in broilers.

## INTRODUCTION

Zinc (Zn) is an essential trace mineral that plays a vital role in numerous biological and physiological functions in broilers, including enzyme activation, immune regulation, antioxidant defense, and skeletal development [1]. Meeting dietary Zn requirements is critical for ensuring optimal growth, health, and bone integrity in modern broiler production systems [2]. Zinc is typically supplemented in poultry diets in either inorganic or organic forms. Inorganic sources, such as zinc oxide and zinc sulfate, are widely used in commercial feeds due to their availability and low cost [3]. However, these sources generally exhibit lower bioavailability than organic Zn sources, especially in corn–soybean meal–based diets. Star et al [2] reported a relative bioavailability of 164% for an organic Zn source, using zinc sulfate as the reference standard in male broilers. Due to the limited absorption of inorganic Zn, diets that meet the total Zn requirement estimates on a concentration basis may still fail to satisfy the birds’ physiological needs [2]. Furthermore, unabsorbed Zn is excreted in feces, contributing to environmental contamination and raising concerns about nutrient management and sustainability [4].

Despite growing interest in organic Zn supplementation, few studies have directly compared the efficacy of organic and inorganic Zn under standardized dietary conditions [5,6]. Additionally, information available in the literature presents inconsistent findings, likely due to variations in bird age, basal diet composition, and the specific chemical forms of Zn evaluated [1]. Few studies have also investigated the optimal supplementation level of organic Zn needed to maximize both growth performance and skeletal development [7,8]. Moreover, due to continuous genetic improvements and advancements in production environments [9], findings from earlier studies that tested the efficacy of organic Zn sources may no longer fully represent contemporary production conditions [10,11]. Therefore, there remains a critical need to refine Zn requirement estimates under contemporary production conditions when using highly bioavailable organic Zn sources. The objectives of this study were to compare the growth performance and bone Zn retention in broilers receiving dietary supplementation with inorganic or organic Zn sources, and to determine the optimal organic Zn concentration to maximize growth performance and skeletal Zn deposition.

## MATERIALS AND METHODS

### Birds, experimental diets, and sample collection

A total of 240 one-d-old Cobb 500 male broiler chicks (initial body weight = 38.4±1.2 g) were randomly assigned to 5 dietary treatments in a completely randomized design. Each treatment was randomly assigned to cages with 8 replicate cages of 6 chicks per cage. From d 0 to 11, birds were fed pre-starter diets, followed by starter diets from d 11 to 20 (Table 1). All diets were formulated to meet or exceed the nutrient requirement estimates for broiler chicks recommended in the Cobb 500 Broiler Nutrition Guide [12] except for Zn. In each phase, a single corn-soybean meal-based basal diet was produced. The analyzed levels of Zn in the basal pre-starter and the starter diets were 32.8 and 31.5 mg/kg, respectively (Table 1). The remaining 4 diets were prepared from the basal diets by supplementing Zn as needed. The 5 dietary treatments were as follows: (1) A basal diet with 40 mg/kg inorganic Zn as ZnSO_4_·7H_2_O; (2) a basal diet without supplemental Zn; (3–5) a basal diet supplemented with 5, 10, or 20 mg/kg of organic Zn as Zn proteinate (Bioplex Zn, Alltech). Birds were housed in battery cages (model SG12; Alternative Design Manufacturing) with free access to feed and water throughout the experiment. Average daily gain (ADG), average daily feed intake (ADFI), and feed conversion ratio (FCR) were determined for the periods of d 0 to 11, 11 to 20, and 0 to 20, after adjusting for mortality. On d 20, 2 birds per cage with body weight closest to the cage median were selected and euthanized via argon asphyxiation. The left tibia from each bird was collected for further analysis.

### Chemical and bone characteristics analysis

Samples of the experimental diets were stored at 4°C before and after grinding and were ground (<0.5 mm) using a centrifugal grinder (ZM 200; Retsch). The ground diets were analyzed for nitrogen (method 990.03), calcium (method 975.03B[b]), and phosphorus (method 966.01; AOAC International [13]) at the Agricultural Experiment Station Chemical Laboratories (University of Missouri). The collected tibias were manually cleaned using a scalpel to remove all attached muscle and soft tissue. The tibias were then extracted with petroleum ether, dried, and ashed overnight in a muffle furnace at 600°C [14]. The resulting ash was weighed and used for Zn analysis. Both ground diet and tibia ash samples were analyzed for Zn concentration (method 975.03B[b]; AOAC International [13]) at the Agricultural Experiment Station Chemical Laboratories.

### Statistical analysis

Data were analyzed using the MIXED procedure of SAS (SAS Institute). Dietary treatment was considered a fixed effect. Outliers, defined as values deviating by more than 1.5 times the interquartile range from the first or third quartile within each dietary treatment group, were assessed but none were identified. Least squares means were calculated for each treatment. Pairwise contrasts were performed between diets containing 40 mg/kg of inorganic Zn and those containing either 10 or 20 mg/kg of organic Zn. Additionally, orthogonal polynomial contrasts were used to evaluate linear and quadratic responses to increasing levels of supplemental organic Zn (0, 5, 10, and 20 mg/kg). Appropriate contrast coefficients for the unequally spaced Zn levels were generated using the IML procedure in SAS. The optimal dietary level of organic Zn for maximizing tibia Zn concentration was estimated using a one-slope broken-line regression model via the NLIN procedure of SAS [15]. A two-sample z-test was performed to compare the predicted Zn concentration in tibia ash at the plateau of the broken-line model with the observed concentration in broilers fed 40 mg/kg of inorganic Zn. The experimental unit was the cage, and statistical significance was declared at p< 0.05.

## RESULTS

**Figure 1:**
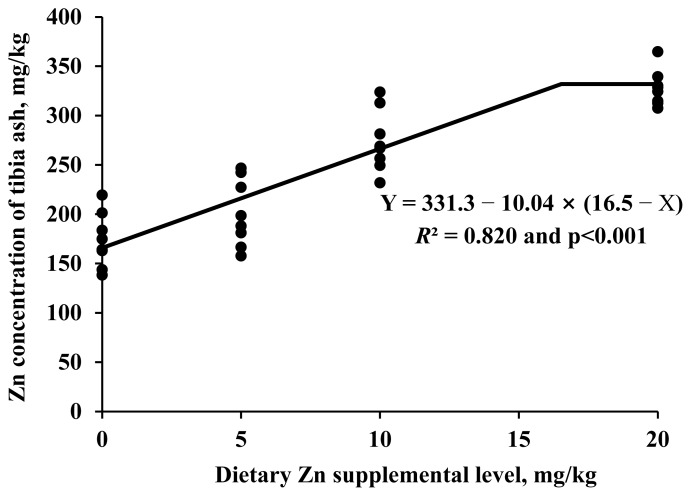
One-slope broken-line analysis of zinc (Zn) concentration in tibia ash in relation to dietary Zn supplementation in broiler chickens (n = 32). The one-slope broken-line model estimated that the minimum dietary Zn required to maximize Zn concentration in tibia ash was 16.5 mg/kg (standard error = 3.2; p<0.001). The break-point was determined using the equation: Zn in tibia ash (mg/kg) = 331.3−10.04×(16.5−X), where X is less than 16.5.

ADFI from d 0 to 11, d 11 to 20, and d 0 to 20 increased linearly (p<0.05) with increasing levels of supplemental organic Zn (Table 2). Similarly, ADG from d 11 to 20 and d 0 to 20 showed a linear increase (p<0.05) as dietary organic Zn increased. Body weight on both d 11 and d 20 were also linearly (p<0.05) increased to increasing level of organic Zn. Tibia Zn weight and concentration increased linearly (p<0.001) with increasing levels of organic Zn supplementation (Table 3). Broilers fed 10 mg/kg of organic Zn had lower (p<0.01) tibia Zn weight and concentrations than those fed 40 mg/kg of inorganic Zn. However, when the organic Zn level was increased to 20 mg/kg, tibia Zn weight and concentrations did not differ from those of birds fed 40 mg/kg of inorganic Zn. Based on the one-slope broken-line regression, tibia Zn concentration plateaued at 327.8 mg/kg (standard error = 10.5) when dietary organic Zn exceeded 16.5 mg/kg (standard error = 3.2; p<0.001; Figure 1), with a linear increase observed below this breakpoint. The predicted tibia Zn concentration at the plateau did not differ (p = 0.332) from the measured value of 341.3 mg/kg in broilers fed 40 mg/kg of inorganic Zn.

## DISCUSSION

The analyzed concentrations of crude protein, calcium, and phosphorus in both pre-starter and starter diets were higher than the calculated values (Table 1), likely due to inherent variability in the nutrient concentrations in the feed ingredients. Despite this, variation among the 5 diets within each phase was minimal, indicating consistency in diet formulation. Linear regression analysis of calculated versus analyzed Zn concentrations in both pre-starter and starter diets revealed a strong positive correlation (Table 1; data not shown; R^2^>0.99 and p<0.001), indicating close agreement between expected and actual Zn levels. This high level of additivity suggests that both inorganic and organic Zn sources were accurately incorporated into the diets as intended.

Zinc is a crucial trace element involved in a wide range of metabolic and physiological processes in broilers, including the activation of metalloenzymes, regulation of immune responses, antioxidant defense mechanisms, and the maintenance of skeletal integrity [1]. The efficiency of Zn utilization largely depends on its dietary source. Organic forms of Zn are generally more bioavailable than their inorganic counterparts, primarily because they are chelated with organic ligands that enhance their stability and solubility in the gastrointestinal tract. This chelation reduces antagonistic interactions with other dietary components that would otherwise form insoluble complexes and limit Zn absorption [16]. Consequently, organic Zn sources exhibit superior intestinal uptake and tissue deposition compared with inorganic Zn salts such as zinc sulfate, as consistently demonstrated in previous study [2]. This improved bioavailability makes organic Zn a more efficient and environmentally sustainable option for meeting the Zn requirements of broilers.

In the present study dietary supplementation of organic Zn resulted in a linear increase in both ADG and ADFI, while FCR did not differ. This indicates that the improvement in ADG was primarily driven by greater ADFI rather than enhanced feed efficiency. These findings are partially consistent with the results summarized in a meta-analysis by Hidayat et al [1], which serves as a broader reference point rather than a direct comparator. The meta-analysis provides supportive evidence that increasing dietary Zn levels enhances ADG (n = 195 and p = 0.008) and improves FCR (n = 154 and p = 0.022), although ADFI was not affected (n = 174 and p = 0.210). The discrepancy in ADFI and FCR outcomes between their study and ours could be attributed to the level of supplemental, the types of Zn sources evaluated, or both. The meta-analysis assessed a wide range of dietary Zn supplementation levels (0 to 200 mg/kg), whereas our study investigated the effects of organic Zn within a relatively narrow range. While our study specifically examined the effects of organic Zn supplementation, their meta-analysis included a broader range of inorganic forms, such as zinc sulfate, zinc carbonate, and zinc oxide, which may have masked source-specific effects. Although zinc sulfate, a highly water-soluble inorganic form, has been shown to negatively affect feed palatability and reduce appetite in chicks [10], the higher ADFI observed in the Zn-supplemented groups in the present study was primarily attributable to the alleviation of Zn deficiency, which enabled birds to consume greater amounts of energy, protein, and other indispensable nutrients, thereby leading to enhanced weight gain [5]. Moreover, increased feed intake also elevates total Zn intake, thereby enhancing the pool of available Zn for absorption and deposition in bone tissue, which may explain the elevated tibia Zn concentration observed in this study. Similar findings were reported by Rao et al [5], who observed increased ADG and ADFI but unchanged FCR in broilers fed 40 mg/kg of Zn proteinate for 21 d. Likewise, Jahanian et al [10] found that, although FCR did not differ between Zn sources, both ADG and ADFI were higher in birds receiving organic Zn compared to those fed inorganic Zn over a 42-d period.

Bone functions as a primary reservoir for minerals, and zinc stored in bone tissue can be mobilized during periods of dietary Zn deficiency [17]. As such, bone Zn content serves as a reliable biomarker of Zn bioavailability in poultry diets [18]. In the present study, Zn concentrations in tibia ash increased linearly with increasing levels of dietary organic Zn supplementation, in agreement with findings from a meta-analysis (n = 96 and p<0.001; Hidayat et al [1]). Although no differences in growth performance were observed among broilers supplemented with 40 mg/kg inorganic Zn and those receiving 10 or 20 mg/kg organic Zn in the current study, tibia Zn weight and concentrations differed among the groups. These findings suggest that 10 mg/kg organic Zn is sufficient to support growth performance equivalent to 40 mg/kg inorganic Zn, but inadequate to maximize bone Zn deposition. In contrast, 20 mg/kg organic Zn appeared to be sufficient for both growth and skeletal Zn requirements. These results highlight the greater Zn demand for optimal bone mineralization compared with that for growth performance, a pattern that has been consistently reported in the literature. For example, Sunder et al [19] demonstrated that supplementing broilers with zinc sulfate at levels ranging from 0 to 320 mg/kg had no effect on ADG, ADFI, or FCR, yet resulted in a linear increase in tibia ash, calcium, and phosphorus concentrations. Similarly, Zhang et al [20] observed no change in growth performance when broilers were supplemented with 0 to 120 mg/kg zinc sulfate during the starter phase; however, tibia ash Zn concentrations increased linearly. Ao et al [11] also reported that supplementation with 40 to 80 mg/kg inorganic Zn or 12 to 24 mg/kg organic Zn had no significant impact on growth performance but led to a linear increase in tibia Zn content. Furthermore, Star et al [2] estimated the relative bioavailability of an organic Zn source to be 164% compared with zinc sulfate, based solely on tibia Zn concentrations, with no differences observed in growth metrics. These findings collectively suggest that the Zn requirement for skeletal development exceeds that for muscle growth, likely due to the continued deposition of Zn in bone tissue even after muscular Zn demands are met. This indicates a greater sensitivity of skeletal tissue to incremental Zn supplementation. A similar physiological distinction has also been documented in swine [21,22].

To determine the optimal dietary organic Zn concentration to maximize tibia Zn deposition, a one-slope broken-line regression analysis was performed [15], a method commonly applied in broiler mineral requirement studies [23]. The analysis showed that tibia Zn concentration reached a plateau at 327.8 mg/kg when dietary organic Zn exceeded 16.5 mg/kg. The 95 percent confidence interval for the breakpoint (10.23 to 22.77 mg/kg) did not include 10 mg/kg, indicating that the Zn concentration required to support optimal bone development may be higher than that required for general growth support. Moreover, the predicted plateau value was not different from the measured tibia Zn concentration of 341.3 mg/kg in broilers fed 40 mg/kg inorganic Zn. This suggests that a lower amount of organic Zn, within the identified range, can support skeletal Zn deposition equivalent to that achieved with a higher level of inorganic Zn. These findings reinforce the greater bioefficacy of organic Zn in supporting bone mineralization.

## CONCLUSION

In conclusion, increasing dietary organic Zn supplementation from d 0 to 20 linearly resulted in benefits to ADG and ADFI, whereas FCR did not differ. To maximize growth performance in broilers, supplementation with 10 mg/kg of organic Zn was sufficient. However, to maximize skeletal development, a higher level of at least 16.5 mg/kg was required. Supplementation with 10 and 16.5 mg/kg of organic Zn effectively replaced 40 mg/kg of inorganic Zn in achieving comparable growth performance and bone Zn deposition in broilers fed a corn-soybean meal-based diet.

## Figures and Tables

**Table 1:** Ingredient and calculated and analyzed chemical composition of experimental diets (as-fed basis)

Item (%)	Phase^1)^	Pre-starter (d 0 to 1^1)^					Starter (d 11 to 20)				
		
	Form of Zn^2)^	Inorganic	Organic				Inorganic	Organic			
				
	Supplemental Zn (mg/kg)	40	0	5	10	20	40	0	5	10	20
Ingredient											
Ground corn		55.67	55.67	55.67	55.67	55.67	60.44	60.44	60.44	60.44	60.44
Soybean meal (48% crude protein)		36.55	36.55	36.55	36.55	36.55	31.72	31.72	31.72	31.72	31.72
Inorganic Zn (mg/kg)		175.7	-	-	-	-	175.7	-	-	-	-
Organic Zn (mg/kg)		-	-	33.3	66.6	133.3	-	-	33.3	66.6	133.3
Vegetable oil		3.30	3.30	3.30	3.30	3.30	3.65	3.65	3.65	3.65	3.65
Limestone		1.13	1.13	1.13	1.13	1.13	1.07	1.07	1.07	1.07	1.07
Dicalcium phosphate		1.74	1.74	1.74	1.74	1.74	1.62	1.62	1.62	1.62	1.62
Sodium chloride		0.41	0.41	0.41	0.41	0.41	0.39	0.39	0.39	0.39	0.39
_L_-Lys·HCl (78.8%)		0.21	0.21	0.21	0.21	0.21	0.18	0.18	0.18	0.18	0.18
_DL_-Met (99.0%)		0.37	0.37	0.37	0.37	0.37	0.33	0.33	0.33	0.33	0.33
_L_-Thr (98.0%)		0.12	0.12	0.12	0.12	0.12	0.10	0.10	0.10	0.10	0.10
Vitamin premix^3)^		0.25	0.25	0.25	0.25	0.25	0.25	0.25	0.25	0.25	0.25
Mineral premix (no Zn)^4)^		0.25	0.25	0.25	0.25	0.25	0.25	0.25	0.25	0.25	0.25
Calculated values											
AME (kcal/kg)		3,050	3,050	3,050	3,050	3,050	3,100	3,100	3,100	3,100	3,100
Crude protein		21.50	21.50	21.50	21.50	21.50	19.50	19.50	19.50	19.50	19.50
Calcium		0.90	0.90	0.90	0.90	0.90	0.84	0.84	0.84	0.84	0.84
Total phosphorus		0.74	0.74	0.74	0.74	0.74	0.69	0.69	0.69	0.69	0.69
Available phosphorus		0.45	0.45	0.45	0.45	0.45	0.42	0.42	0.42	0.42	0.42
SID Lys		1.19	1.19	1.19	1.19	1.19	1.02	1.02	1.02	1.02	1.02
SID Met		0.64	0.64	0.64	0.64	0.64	0.58	0.58	0.58	0.58	0.58
SID Met+Cys		0.90	0.90	0.90	0.90	0.90	0.82	0.82	0.82	0.82	0.82
SID Thr		0.78	0.78	0.78	0.78	0.78	0.70	0.70	0.70	0.70	0.70
Analyzed values											
Zn (mg/kg)		79.9	32.8	37.0	42.7	52.1	65.7	31.5	37.1	42.0	52.2
Crude protein		23.25	22.63	22.13	22.50	22.56	20.69	20.44	20.75	20.81	20.75
Calcium		1.26	1.23	1.21	1.27	1.30	1.07	1.18	1.16	1.27	1.15
Phosphorus		0.90	0.90	0.89	0.94	0.91	0.74	0.84	0.83	0.87	0.86

1) Pre-starter and starter diets were fed for d 0 to 11 and from d 11 to 20, respectively.

2) The forms of inorganic and organic Zn used were ZnSO_4_·7H_2_O (227.7 g of Zn/kg of Zn sulfate heptahydrate) and Bioplex Zn (150 g of Zn/kg of Bioplex Zn), respectively.

3) The vitamin premix provided the following quantities per kilogram of complete diet: vitamin A as retinyl acetate, 11,026 IU; vitamin D_3_ as cholecalciferol, 3,528 IU; vitamin E as _DL_-α-tocopheryl acetate, 36.3 IU; vitamin K_3_ as menadione, 0.91 mg; thiamin, 2 mg; riboflavin, 8 mg; niacin, 55 mg; _D_-pantothenic acid as _D_-calcium pantothenate, 18 mg, pyridoxine, 5 mg, biotin, 0.221 mg; folic acid, 1 mg; choline as choline chloride, 480 mg; and vitamin B_12_ as cyanocobalamin, 0.028 mg.

4) The mineral premix provided the following quantities per kilogram of complete diet: iron as ferrous sulfate, 40 mg; manganese as manganese sulfate, 100 mg; copper as cupric sulfate, 15 mg; and selenium as sodium selenite, 0.20 mg.

Zn, zinc; AME, apparent metabolizable energy; SID, standardized ileal digestible.

**Table 2:** Effects of inorganic and organic zinc supplementation on growth performance in broiler chickens

Item	Form of zinc	Inorganic	Organic				SEM	p-values for			

								Orthogonal contrast^1)^		Polynomial contrast^2)^	
		
	Supplemental zinc (mg/kg)	40	0	5	10	20		40 vs. 10	40 vs. 20	Linear	Quadratic
d 0 to 11											
Initial BW (g/bird)		38.3	38.0	38.6	38.5	38.1	0.4	0.723	0.723	0.918	0.153
ADG (g/bird/d)		21.1	20.9	20.6	21.4	22.0	0.7	0.736	0.335	0.164	0.745
ADFI (g/bird/d)		26.2	24.7	24.5	26.2	26.0	0.5	0.990	0.800	0.038	0.481
Feed conversion ratio (g/g)		1.25	1.19	1.19	1.22	1.18	0.03	0.578	0.120	0.991	0.261
Final BW (g/bird)		270.6	268.2	265.3	274.3	286.1	6.9	0.711	0.123	0.046	0.555
d 11 to 20											
ADG (g/bird/d)		61.1	56.3	55.2	59.3	60.0	1.4	0.359	0.576	0.024	0.964
ADFI (g/bird/d)		79.7	75.6	74.0	78.6	78.9	1.3	0.567	0.656	0.029	0.929
Feed conversion ratio (g/g)		1.31	1.34	1.34	1.33	1.32	0.02	0.406	0.718	0.203	0.995
Final BW (g/bird)		835.7	775.3	775.9	807.6	825.8	15.3	0.203	0.651	0.014	0.967
d 0 to 20											
ADG (g/bird/d)		39.0	36.9	36.1	38.5	38.9	0.7	0.555	0.860	0.015	0.975
ADFI (g/bird/d)		50.2	47.6	46.7	49.8	49.5	0.6	0.676	0.465	0.012	0.622
Feed conversion ratio (g/g)		1.29	1.29	1.29	1.29	1.27	0.01	0.628	0.496	0.238	0.482

The experimental duration was 20 d. Each least squares mean represents 8 replicate cages pet treatment and 6 birds per cage.

1) Pairwise orthogonal contrasts were performed between diets containing 40 mg/kg of inorganic zinc and those containing either 10 or 20 mg/kg of organic zinc.

2) Orthogonal polynomial contrasts were used to evaluate linear and quadratic responses to increasing levels of supplemental organic zinc (0, 5, 10, and 20 mg/kg).

SEM, standard error of the mean; BW, body weight; ADG, average daily gain; ADFI, average daily feed intake.

**Table 3:** Effects of inorganic and organic zinc supplementation on tibia growth and mineralization parameters in broiler chickens

Item	Form of zinc	Inorganic	Organic				SEM	p-values for			

								Orthogonal contrast^1)^		Polynomial contrast^2)^	
				
	Supplemental zinc (mg/kg)	40	0	5	10	20		40 vs. 10	40 vs. 20	Linear	Quadratic
Moisture in fresh tibia (%)		57.6	57.5	58.4	57.5	57.1	0.5	0.806	0.425	0.176	0.334
Fat in dried tibia (%)		3.50	3.88	3.29	3.98	3.87	0.58	0.555	0.650	0.811	0.848
Fat-free tibia ash (%)		52.5	52.7	53.2	52.2	53.4	0.6	0.727	0.348	0.636	0.525
Zinc weight in tibia (mg)		0.285	0.140	0.167	0.231	0.266	0.011	0.002	0.244	<0.001	0.185
Zinc concentration in tibia ash (mg/kg)		341.3	173.5	201.0	273.9	327.8	9.2	<0.001	0.303	<0.001	0.264

Each least squares mean represents 8 replicate cages per treatment and 2 birds per cage.

1) Pairwise orthogonal contrasts were performed between diets containing 40 mg/kg of inorganic zinc and those containing either 10 or 20 mg/kg of organic zinc.

2) Orthogonal polynomial contrasts were used to evaluate linear and quadratic responses to increasing levels of supplemental organic zinc (0, 5, 10, and 20 mg/kg).

SEM, standard error of the mean.

## Data Availability

Upon reasonable request, the datasets of this study can be available from the corresponding author.
